# Correlates of childhood morbidity in Nigeria: Evidence from ordinal analysis of cross-sectional data

**DOI:** 10.1371/journal.pone.0233259

**Published:** 2020-05-14

**Authors:** Sulaimon T. Adedokun

**Affiliations:** Department of Demography and Social Statistics, Obafemi Awolowo University, Ile-Ife, Nigeria; The University of Hong Kong, CHINA

## Abstract

**Background:**

Child mortality records show that 1 in every 13 children dies before age five in sub-Saharan Africa with diseases such as pneumonia, diarrhoea and malaria considered to be the leading causes of such deaths. In Nigeria where 50% of all under-five deaths are attributed to morbidity, much attention has been directed to single health conditions. This study aims at examining the factors that are associated with single health conditions and comorbidity among children in Nigeria.

**Materials and methods:**

This study was based on data from 2013 Nigeria Demographic and Health Survey (DHS) which involved 27,571 under-five children who suffered from acute respiratory infection, diarrhoea or fever within two weeks of data collection exercise. Descriptive statistics and generalized ordinal logistic regression model were used for the analysis.

**Results:**

About 14% of children suffered from a single health condition and 9% suffered from comorbidity. The likelihood of suffering from a single health condition and comorbidity is higher for children who are of third order birth or more (OR = 1.24, 95% CI = 1.11–1.39 & OR = 1.31, 95% CI = 1.12–1.55) compared to those who are of first order birth. The likelihood also increased for children whose mothers live in Northeast (OR = 3.19, 95% CI = 2.86–3.55 & OR = 3.88, 95% CI = 3.30–4.57) compared to children whose mothers live in North Central. The odds of suffering from a single health condition and comorbidity reduced for children who are from richest households, aged 3 years and above and were of average size at birth. Children of women who obtained water from improved source are less likely to experience any morbidity (OR = 0.93, 95% CI = 0.87–0.99) compared to children whose mothers obtained water from non-improved source.

**Conclusions:**

The study has demonstrated that children in Nigeria are not only exposed to the risk of single health conditions but they are also exposed to the risk of comorbidity. Efforts should be made to design appropriate health care models that would facilitate a considerable reduction in childhood morbidity in the country.

## Introduction

Child mortality records show that in 2017, 1 in every 13 children died before age 5 in sub-Saharan Africa [1]. While the region is also responsible for 50% of all under-five deaths in the world, diseases such as pneumonia, diarrhoea and malaria are considered to be the leading causes of such deaths [1]. Different initiatives have been introduced to reduce the number of children that would suffer from these diseases and at the same time ensure that children who have been affected by the diseases are properly treated. The World Health Organization and UNICEF introduced Integrated Management of Childhood Illness in 1996 in order to manage diarrhea and other major diseases that lead to mortality [2]. The Integrated Global Action for the Prevention and Control of Pneumonia and Diarrhoea (GAPPD) was launched with the objective of providing a framework for interventions that would protect children’s health, prevent disease and treat children who have been diagnosed with diarrhoea and pneumonia [3]. The objective was anchored on two components: (i) protective interventions which aimed at keeping children healthy and free from disease through exclusive breastfeeding, adequate complementary feeding and continued breastfeeding and vitamin A supplementation and; (ii) preventive interventions which focused on prevention of disease transmission and prevention of children from becoming sick through immunization and safe drinking water, sanitation and hygiene [3]. The Roll Back Malaria was also established in 1998 for the purpose of creating a stronger action to curb malaria [4]. To ensure that this action is well consolidated, Global Technical Strategy for Malaria was adopted by WHO Assembly in 2015. The strategy aimed at providing a guide for countries in the fight against malaria and setting a target of reducing malaria incidence and mortality by 90% by 2030 [5].

The situation of childhood morbidity in Nigeria is not different from what obtains at sub-Saharan African level. Morbidity contributes significantly to child mortality in the country as diarrhoea, malaria and lower respiratory infection have been described as being among the top ten causes of death in 2017 [6]. The country also adopted programmes and initiatives of WHO and UNICEF in combating childhood diseases. Some of the efforts made in this regard include (i) ensuring that at least 60% of those at risk of malaria, particularly under-five children, receive and use insecticide treated nets; (ii) ensuring correct diagnosis and treatment of childhood diseases, prompt referral and promoting preventive measures at home, communities and health facility and; (iii) ensuring reduction in malnutrition among under-five children [7]. Despite these efforts, childhood morbidity is still a major challenge in the country. While malaria accounts for 30% of admission into children emergency ward, 20% and 19% are admitted for diarrhoea and respiratory tract infections respectively [8]. For this reason, close to 50% of under-five deaths are attributed to morbidity [9]. Factors that influence childhood morbidity have been described in different studies. Such factors include maternal education, household wealth, immunization, water source, toilet facility, dirty surroundings, source of cooking fuel, indoor pollution, level of hygiene practice, etc. [10–17]. However, most of these studies focused more on single health condition while paying little attention to comorbidity. Knowledge about prevalence of single health conditions and comorbidity among children together with a better understanding of the factors associated with such conditions is needed in order to identify appropriate interventions that would lead to the design and application of appropriate health care model. This study aims at filling this gap by examining the factors influencing single health conditions and comorbidity among children in Nigeria in a single analytical framework.

## Materials and methods

### Study design

The data used for analysis in this study were obtained from 2013 Nigeria Demographic and Health Survey (NDHS). The survey, which is cross-sectional, was conducted in all the states and regions of the country. It provides adequate information on health and other related issues about the population.

### Sampling technique

Sample for the survey was selected using a multi-stage cluster sampling technique. The country was divided into 37 units which included the Federal Capital Territory (FCT). Out of these units, 896 communities were selected based on the primary sampling unit (PSU) method adopted during the last census exercise. About 904 clusters were later generated from these communities with 372 and 532 in urban and rural areas respectively. A total sample of 40,680 households was finally selected for the survey. All women within the reproductive age of 15–49 who were either residents of the households or visitors present in the households a night before the survey were considered for interview.

### Data collection

A model questionnaire designed by MEASURE DHS programme was used to obtain information from women aged 15–49 years through face-to-face interview. The women were asked questions relating to background characteristics, reproductive history, antenatal, delivery and postnatal care, childhood immunization and diseases. Details of data collection procedure have been published elsewhere [18].

### Outcome variable

The study considered children under the age of 5 who had fever, diarrhoea or acute respiratory infection in two weeks preceding the survey. Mothers were asked about the symptoms of each of these illnesses in their children. For instance, acute respiratory infection was measured by asking mothers if their children had been ill with a cough accompanied by short, rapid breathing. The outcome variable was obtained from the three variables representing the illnesses. Children who did not suffer from any of the illnesses were categorised as none, those who suffered from one illness were defined as one, those who suffered from two illnesses were categorised as two and those who suffered from all the illnesses were categorised as three. For easy interpretation, children who experienced one type of illness were referred to as having single health condition while those who experienced two or all the illnesses were referred to as having comorbidity.

### Independent variables

We considered the following independent variables in the study: mother’s age, education, household wealth, residence, region, sex of child and child’s current age. Others include media exposure, child’s size at birth, birth order, place of delivery, source of water and method of cooking. Mother’s age was categorised into 15–24, 25–34 and 35+. Education was defined as no education, primary and secondary/higher. Household wealth was grouped into five quintiles namely poorest, poorer, middle, richer and richest. Residence was defined in terms of urban and rural. Region was categorised into North Central, North east, North west, South east, South South and South west. Sex of child was grouped into male and female. Child’s current age was categorised into less than 1 year, 1–2 years and 3 years and above. Media exposure was dichotomised as not exposed (for those who did not use any of the news outlets) and exposed (for those who used at least one of the news outlets). Child’s size at birth was measured as large, average and small. Birth order was defined as 1^st^ order, 2^nd^ order and 3^rd^ order and above. Place of delivery was grouped into outside health facility and health facility. Source of water was defined as not improved and improved. Method of cooking was also measured as not improved and improved.

### Statistical analysis

Analysis in this study was carried out in two stages. The first stage involved descriptive analysis which presents the distribution of respondents’ characteristics against the outcome variable. In order to adjust for under-reporting and over-reporting the survey, weighting factor defined as “$\left(\frac{v005}{1000000}\right)$”, where *v*005 is the sample weight, was applied to the data using *svyset* command. The second stage involved the application of an ordinal logistic regression model based on the nature of the outcome variable. Ordinal logistic regression is applied when the outcome variable has categories that are ordered. The outcome variable in this study has four ordered categories namely none, one, two and three. The model is premised on the proportional odds assumption which assumes that each independent variable has the same effects across the categories of outcome variable [19]. In order to ascertain the appropriateness of the application of this model to the study, proportional odds assumption was tested through Brant method. In view of this violation of proportionality assumption, generalized ordinal logistic regression modelling was applied. This model relaxes the proportional odds assumption by allowing the effect of all independent variables to vary across the categories of outcomes. The generalized ordinal logistic regression model is given as:

$$logit\left[\pi \left(Y>j\text{|}x_{1},x_{2},\ldots ,x_{p}\right)\right]=In\;\left(\frac{\pi \left(Y>j|x_{1},x_{2},\ldots ,x_{p}\right)}{\pi \left(Y\leq J|x_{1},x_{2},\ldots ,x_{p}{}_{1}\right)}\right)=\alpha_{j}+(\beta_{j1}X_{1}+\beta_{2j}X_{2}+\ldots +\beta_{pj}X_{p})$$

Where *αj* represents the intercepts and *βj*1, … *βjp* represent the logit coefficients; and *X*_1_…*X_p_* represent the independent variables [19]. The model estimates the odds of being beyond a certain category in relation to being at or below the category [19]. Odds ratios (ORs) were obtained and interpreted in respect of single health conditions and comorbidity. In order to have a robust explanation for the relationship between independent variables and the outcome variable, 𝛼 level of 0.05 was specified. Stata statistical software version 14 was used to perform all statistical operations.

### Ethics approval and consent to participate

The survey was approved by Institutional Review Board (IRB) of ICF Macro International in the United States and the National Ethics Committee in the Federal Ministry of Health of Nigeria. National Population Commission obtained both oral and written consent from the participants in the survey.

## Results

Four tables were used to present the results. Table 1 shows results of the test of proportional odds assumption. The Brant test *X*^2^ = 225.85, p = 0.000 implies that the proportional odds assumption in respect of the overall model has been violated. Considering each independent variable, it was found that age, place of delivery, source of water and method of cooking met the proportional odds assumption while education, household wealth index, region, child’s current age, media exposure, child’s size at birth and birth order violated this assumption. In the second table, results were expressed in number and percentages. The results showed the relationship between independent variables and specific illnesses. In the third table, results were expressed in number and percentages including the p-value obtained from Chi-Square test. Only the variables found to be significant at this stage were included in the model.

**Table 1:** Results of brant test for proportional odds assumption.

	*X*^2^	p-value
Overall Value	225.85	0.000
Variables		
Age		
25–34	0.24	0.885
35+	1.01	0.603
Education		
Primary	4.38	0.122
Secondary/Higher	10.76	0.005
Household wealth index		
Poorer	4.29	0.117
Middle	9.12	0.010
Richer	8.03	0.018
Richest	2.43	0.297
Region		
North East	11.43	0.003
North West	3.45	0.178
South East	3.58	0.167
South South	12.15	0.002
South West	13.29	0.001
Child’s current age		
1–2 years	3.80	0.149
3+ years	20.36	0.000
Media Exposure		
Exposed	9.45	0.009
Child’s size at birth		
Average	5.96	0.051
Small	8.88	0.012
Birth order		
2^nd^ order	7.06	0.029
3^rd^ order	0.98	0.614
Place of delivery		
Health facility	4.07	0.131
Source of water		
Improved	3.41	0.182
Method of cooking		
Improved	2.63	0.268

### Descriptive statistics

Tables 2 and 3 show the results of descriptive analysis. In Table 2, it is revealed that about 11% of the children had diarrhoea, 13% had fever and 10% had acute respiratory infection (ARI). Highest prevalence of morbidity is recorded among children of women who are between 15 and 24 years of age. Among this category of women, 13% of children suffered from diarrhoea, 14% suffered from fever and 11% suffered from ARI. With respect to education, most children suffering from diarrhoea (13%) are from uneducated women while most children who suffered from fever (14%) and ARI (13%) are from women with primary and secondary or higher education respectively. Majority of the children with diarrhoea (14%) and fever (15%) are from poorest households while most of the children with ARI (11%) are from middle wealth households. The region with the highest prevalence of childhood morbidity is Northeast. In this region, 22% of the children suffered from diarrhoea and fever while 17% suffered from ARI. While diarrhoea is common among infants (12%), fever (16%) and ARI (12%) are common among children who are between 1 and 2 years of age. Child morbidity is highest among children who were small at birth. Among this category of children, 15% suffered from diarrhoea, 16% suffered from fever and 11% suffered from ARI. In a similar vein, children of 3^rd^ order of birth or higher experienced highest morbidity. While 13% of these children experienced diarrhoea, 15% experienced fever. Also, 12% of children of women who obtain water from non-improved source suffered from diarrhoea. While 14% of children in this category suffered from fever, 11% suffered from ARI. Table 3 shows that 14% of the children suffered from a single health condition, 7% suffered from two health conditions and 2% suffered from all the three health conditions. While about 15% of the children of women aged 15–24 experienced a single health condition, less than 10% experienced comorbidity (8% for 2 conditions and 3% for 3 conditions). Although the highest percentage of children suffering from the three illnesses is found among women with no education (3%), women with primary and secondary or higher education have the highest percentage of children suffering from two illnesses (7%). Most of the children of women from poorest and poorer households experienced single health condition (15% and 16% respectively). Comorbidity is also common among children in these households. Northeast is the region with highest percentage of children experiencing single health condition (20%) and comorbidity (12% for two conditions and 6% for three conditions). About 17% of children aged 1–2 years and 15% of those whose mothers were not exposed to media suffered from single health condition. Morbidity is most prevalent among children who were small at birth as 18% and 11% of such children experienced single health condition and comorbidity respectively.

**Table 2:** Descriptive analysis of specific childhood morbidity in Nigeria.

Variables	Childhood morbidity					
	Diarrhoea		Fever		ARI	
	No	Yes	No	Yes	No	Yes
	N (%)	N (%)	N (%)	N (%)	N (%)	N (%)
	24,647 (89.4)	2,924 (10.6)	23,951(86.9)	3,620 (13.1)	24,808 (90.0)	2,763 (10.0)
Mother’s age						
15–24	5,699 (87.3)	831 (12.7)	5,620 (86.1)	910 (13.9)	5,799 (88.8)	731 (11.2)
25–34	12,499 (89.9)	1,407 (10.1)	12,107 (87.1)	1,799 (12.9)	12,526 (90.1)	1,380 (9.9)
35+	6,449 (90.4)	686 (9.6)	6,224 (87.2)	911 (12.8)	6,483 (90.9)	652 (9.1)
Education						
No education	11,080 (87.3)	1,611 (12.7)	11,005 (86.7)	1,686 (13.3)	11,739 (92.5)	952 (7.5)
Primary	5,039 (89.6)	583 (10.4)	4,847 (86.2)	775 (13.8)	4,976 (88.5)	646 (11.5)
Secondary/higher	8,528 (92.1)	730 (7.9)	8,099 (87.5)	1,159 (12.5)	8,093 (87.4)	1,165 (12.6)
Household wealth index						
Poorest	5,210 (86.3)	829 (13.7)	5,162 (85.5)	877 (14.5)	5,531 (91.6)	508 (8.4)
Poorer	5,476 (87.4)	792 (12.6)	5,368 (85.6)	900 (14.4)	5,644 (90.0)	624 (10.0)
Middle	4,957 (89.9)	561 (10.2)	4,729 (85.7)	789 (14.3)	4,900 (88.8)	618 (11.2)
Richer	4,746 (91.6)	434 (8.4)	4,572 (88.3)	608 (11.7)	4,666 (90.1)	514 (9.9)
Richest	4,258 (93.3)	308 (6.7)	4,120 (90.2)	446 (9.8)	4,067 (89.1)	499 (10.9)
Residence						
Urban	8,520 (90.7)	871 (9.3)	8,233 (87.7)	1,158 (12.3)	8,381 (89.3)	1,010 (10.7)
Rural	16,127 (88.7)	2,053 (11.3)	15,718 (86.5)	2,462 (13.5)	16,427 (90.4)	1,753 (9.6)
Region						
North Central	3,849 (92.9)	293 (7.1)	3,832 (92.5)	310 (7.5)	3,811 (92.0)	331 (8.0)
North East	4,382 (77.9)	1,246 (22.1)	4,395 (78.1)	1,233 (21.9)	4,688 (83.3)	940 (16.7)
North West	7,715 (90.7)	789 (9.3)	7,641 (89.9)	863 (10.1)	8,135 (95.7)	369 (4.3)
South East	2,236 (90.3)	240 (9.7)	1,978 (79.9)	498 (20.1)	2,097 (84.7)	379 (15.3)
South South	3,194 (96.1)	130 (3.9)	2,860 (86.0)	464 (14.0)	2,851 (85.8)	473 (14.2)
South West	3,271 (93.5)	226 (6.5)	3,245 (92.8)	252 (7.2)	3,226 (92.3)	271 (7.7)
Sex of child						
Male	12,433 (89.4)	1,472 (10.6)	12,039 (86.6)	1,866 (13.4)	12,493 (89.9)	1,412 (10.1)
Female	12,214 (89.4)	1,452 (10.6)	11,912 (87.2)	1,754 (12.8)	12,315 (90.1)	1,351 (9.9)
Child’s current age						
<1 year	5,488 (88.5)	715 (11.5)	5,424 (87.4)	779 (12.6)	5,522 (89.0)	681 (11.0)
1–2 years	9,372 (85.9)	1,545 (14.1)	9,145 (83.8)	1,772 (16.2)	9,587 (87.8)	1,330 (12.2)
3+ years	9,787 (93.7)	664 (6.3)	9,382 (89.9)	1,069 (10.2)	9,699 (92.8)	752 (7.2)
Media exposure						
Never exposed	8,248 (86.6)	1,281 (13.4)	8,166 (85.7)	1,363 (14.3)	8,586 (90.1)	943 (9.9)
Exposed	16,399 (90.9)	1,643 (9.1)	15,785 (87.5)	2,257 (12.5)	16,222 (89.9)	1,820 (10.1)
Child’s size at birth						
Large	10,791 (89.0)	1,331 (11.0)	10,500 (86.6)	1,622 (13.4)	10,819 (89.2)	1,303 (10.8)
Average	10,164 (90.9)	1,014 (9.1)	9,828 (87.9)	1,350 (12.1)	10,185 (91.1)	993 (8.9)
Small	3,313 (85.5)	561 (14.5)	3,261 (84.2)	613 (15.8)	3,431 (88.6)	443 (11.4)
Birth order						
1^st^ order	12,758 (90.1)	1,397 (9.9)	12,387 (87.5)	1,768 (12.5)	12,691 (89.7)	1,464 (10.3)
2^nd^ order	7,916 (89.2)	959 (10.8)	7,692 (86.7)	1,183 (13.3)	8,038 (90.6)	837 (9.4)
3^rd^ order and above	3,973 (87.5)	568 (12.5)	3,872 (85.3)	669 (14.7)	4,079 (89.8)	462 (10.2)
Place of delivery						
Outside health facility	14,997 (87.9)	2,063 (12.1)	14,719 (86.3)	2,341 (13.7)	15,507 (90.9)	1,553 (9.1)
Health facility	9,503 (91.8)	850 (8.2)	9,084 (87.7)	1,269 (12.3)	9,155 (88.4)	1,198 (11.6)
Source of water						
Not improved	10,778 (88.2)	1,440 (11.8)	10,508 (86.0)	1,710 (14.0)	10,933 (89.5)	1,285 (10.5)
Improved	13,650 (90.3)	1,465 (9.7)	13,232 (87.5)	1,883 (12.5)	13,669 (90.4)	1,446 (9.6)
Method of cooking						
Not improved	23,998 (89.3)	2,885 (10.7)	23,335 (86.8)	3,548 (13.2)	24,205 (90.0)	2,678 (10.0)
Improved	460 (96.8)	15 (3.2)	431 (90.7)	44 (9.3)	424 (89.3)	51 (10.7)

**Table 3:** Descriptive analysis of factors associated with childhood morbidity in Nigeria.

Variable	Childhood morbidity status					
	None	One	Two	Three	Total	P value
	N (%)	N (%)	N (%)	N (%)	N (%)	
	21,259 (77.1)	3,918 (14.2)	1,793 (6.5)	601 (2.2)	27,571 (100.0)	
Mother’s age						
15–24	4,879 (74.7)	995 (15.2)	491 (7.5)	165 (2.5)	6,530 (100.0)	
25–34	10,777 (77.5)	1,956 (14.1)	889 (6.4)	284 (2.0)	13,906 (100.0)	
35+	5,603 (78.5)	967 (13.6)	413 (5.8)	152 (2.1)	7,135 (100.0)	<0.001
Education						
No education	9,818 (77.4)	1,816 (14.3)	738 (5.8)	319 (2.5)	12,691 (100.0)	
Primary	4,281 (76.2)	809 (14.4)	401 (7.1)	131 (2.3)	5,622 (100.0)	
Secondary/higher	7,160 (77.3)	1,293 (14.0)	654 (7.1)	151 (1.6)	9,285 (100.0)	<0.001
Household wealth index						
Poorest	4,545 (75.3)	925 (15.3)	418 (6.9)	151 (2.5)	6,039 (100.0)	
Poorer	4,704 (75.1)	980 (15.6)	416 (6.6)	168 (2.7)	6,268 (100.0)	
Middle	4,221 (76.5)	764 (13.9)	395 (7.2)	138 (2.5)	5,518 (100.0)	
Richer	4,113 (79.4)	670 (12.9)	305 (5.9)	92 (1.8)	5,180 (100.0)	
Richest	3,676 (80.5)	579 (12.7)	259 (5.7)	52 (1.1)	4,566 (100.0)	<0.001
Residence						
Urban	7,302 (77.8)	1,318 (14.0)	592 (6.3)	179 (1.9)	9,391 (100.0)	
Rural	13,957 (76.8)	2,600 (14.3)	1,201 (6.6)	422 (2.3)	18,180 (100.0)	0.077
Region						
North Central	3,473 (83.9)	450 (10.9)	173 (4.2)	46 (1.1)	4,142 (100.0)	
North East	3,516 (62.5)	1,136 (20.2)	645 (11.5)	331 (5.9)	5,628 (100.0)	
North West	7,010 (82.4)	1,055 (12.4)	351 (4.1)	88 (1.0)	8,504 (100.0)	
South East	1,737 (70.2)	435 (17.6)	230 (9.3)	74 (3.0)	2,476 (100.0)	
South South	2,614 (78.6)	397 (11.9)	269 (8.1)	44 (1.3)	3,324 (100.0)	
South West	2,909 (83.2)	445 (12.7)	125 (3.6)	18 (1.0)	3,497 (100.0)	<0.001
Sex of child						
Male	10,672 (76.8)	2,025 (14.6)	899 (6.5)	309 (2.2)	13,905 (100.0)	
Female	10,587 (77.5)	1,893 (13.9)	894 (6.5)	292 (2.1)	13,666 (100.0)	0.360
Child’s current age						
<1 year	4,766 (76.8)	859 (13.9)	418 (6.7)	160 (2.6)	6,203 (100.0)	
1–2 years	7,823 (71.7)	1,867 (17.1)	901 (8.3)	326 (3.0)	10,917 (100.0)	
3+ years	8,670 (83.0)	1,192 (11.4)	474 (4.5)	115 (1.1)	10,451 (100.0)	<0.001
Media exposure						
Never exposed	7,179 (75.3)	1,406 (14.8)	651 (6.8)	293 (3.1)	9,529 (100.0)	
Exposed	14,080 (78.0)	2,512 (13.9)	1,142 (6.3)	308 (1.7)	18,042 (100.0)	<0.001
Child’s size at birth						
Large	9,282 (76.6)	1,718 (14.2)	828 (6.8)	294 (2.4)	12,122 (100.0)	
Average	8,855 (79.2)	1,487 (13.3)	638 (5.7)	198 (1.8)	11,178 (100.0)	
Small	2,781 (71.8)	676 (17.5)	310 (8.0)	107 (2.8)	3,874 (100.0)	<0.001
Birth order						
1^st^ order	11,012 (77.8)	1,931 (13.6)	938 (6.6)	274 (1.9)	14,155 (100.0)	
2^nd^ order	6,814 (76.8)	1,339 (15.1)	526 (5.9)	196 (2.2)	8,875 (100.0)	
3^rd^ order and above	3,433 (75.6)	648 (14.3)	329 (7.3)	131 (2.9)	4,541 (100.0)	<0.001
Place of delivery						
Outside health facility	13,070 (76.6)	2,443 (14.3)	1,127 (6.6)	420 (2.5)	17,060 (100.0)	
Health facility	8,053 (77.8)	1,463 (14.1)	657 (6.4)	180 (1.7)	10,353 (100.0)	<0.001
Source of water						
Not improved	9,210 (75.4)	1,875 (15.4)	839 (6.9)	294 (2.4)	12,218 (100.0)	
Improved	11,863 (78.5)	2,015 (13.3)	932 (6.2)	305 (2.0)	15,115 (100.0)	<0.001
Method of cooking						
Not improved	20,709 (77.0)	3,832 (14.3)	1,747 (6.5)	595 (2.2)	26,883 (100.0)	
Improved	390 (82.1)	62 (13.1)	21 (4.4)	2 (0.0)	475 (100.0)	<0.001

### Factors associated with childhood morbidity

Results in Table 4 show that the odds of a child having at least one health condition (1, 2 or 3 illnesses) is 0.88 for women aged 25–34 and 0.78 for women aged 35 years and above. Similarly, the odds of a child experiencing comorbidity (2 or 3 illnesses) is 0.78 for women aged 35 years and above. Children of women who have primary and secondary or higher education are 20% and 30% respectively more likely to suffer from at least one health condition compared to children of women with no education. Also, children of women with primary and secondary or higher education are 29% and 42% respectively more likely to experience comorbidity. Children of women from richest households have 0.76 odds of suffering from at least one health condition and 0.85 odds of experiencing comorbidity. With respect to region, the probability of experiencing at least one health condition increased 3.2 times for children in Northeast, 2.2 times for children in Southeast and 1.4 times for children in South-South. The probability of experiencing comorbidity increased 3.9 times for children in Northeast, 2.5 times for children in Southeast and 1.8 times for children in South-South. While children who are in within the age range 1–2 years are 36% and 27% more likely to experience at least one health condition and comorbidity respectively, children who are 3 years and above are 28% and 39% less likely to experience at least one health condition and comorbidity respectively. The odds of experiencing at least one health condition reduced by 19% for children who were of average size at birth while it increased by 15% for children were small at birth. The odds of experiencing comorbidity also reduced by 26% for children who were of average size at birth compared to children who were large at birth. While children of 2^nd^ order of birth are 16% more likely to experience at least one health condition, children of 3^rd^ order of birth and above are 24% and 31% more likely to experience at least one health condition and comorbidity respectively. Children of women who obtain water from improved source are 6% less likely to experience at least one health condition. The odds of experiencing all the health conditions increased for children from middle wealth households (aOR = 1.42, 95% CI = 1.10–1.85), Northeast (aOR = 5.34, 95% CI = 3.86–7.39) and Southeast (aOR = 3.17, 95% CI = 2.15–4.66). However, the odds reduced for children who are 3 years and above (aOR = 0.43, 95% CI = 0.34–0.55), whose mothers are exposed to media (aOR = 0.82, 95% CI = 0.67–0.99) and who are of average size at birth (aOR = 0.68, 95% CI = 0.57–0.82).

**Table 4:** Results of generalized ordinal logistic regression for childhood morbidity in Nigeria.

Variables	Childhood morbidity		
	(One, two & three) versus none	(Two & three) versus one & none	Three versus (none, one & two)
Mother’s age	aOR (95% CI)	aOR (95% CI)	aOR (95% CI)
15–24	1	1	1
25–34	0.88** (0.81–0.96)	0.91 (0.81–1.03)	0.95 (0.75–1.19)
35+	0.78*** (0.70–0.88)	0.78** (0.66–0.92)	0.97 (0.70–1.33)
Education			
No education	1	1	1
Primary	1.20*** (1.10–1.31)	1.29*** (1.13–1.46)	1.06 (0.84–1.33)
Secondary/higher	1.30*** (1.18–1.44)	1.42*** (1.23–1.65)	0.97 (0.73–1.28)
Household wealth index			
Poorest	1	1	1
Poorer	1.04 (0.95–1.13)	1.02 (0.90–1.16)	1.25 (0.99–1.57)
Middle	0.97 (0.87–1.08)	1.07 (0.92–1.24)	1.42** (1.10–1.85)
Richer	0.80*** (0.71–0.90)	0.83* (0.70–0.99)	1.23 (0.89–1.70)
Richest	0.76*** (0.66–0.88)	0.85 (0.69–1.04)	1.06 (0.70–1.58)
Region			
North Central	1	1	1
North East	3.19*** (2.86–3.55)	3.88*** (3.30–4.57)	5.34*** (3.86–7.39)
North West	1.14* (1.02–1.27)	1.01 (0.84–1.21)	0.90 (0.62–1.32)
South East	2.23*** (1.96–2.53)	2.46*** (2.03–2.97)	3.17*** (2.15–4.66)
South South	1.39*** (1.23–1.57)	1.75*** (1.45–2.11)	1.27 (0.84–1.94)
South West	1.12 (0.99–1.28)	0.80 (0.64–1.00)	0.57* (0.32–0.99)
Child’s current age			
<1 year	1	1	1
1–2 years	1.36*** (1.26–1.47)	1.27*** (1.14–1.41)	1.18 (0.98–1.43)
3+ years	0.72*** (0.66–0.78)	0.61*** (0.54–0.70)	0.43*** (0.34–0.55)
Media exposure			
Never exposed	1	1	1
Exposed	1.10* (1.11–1.53)	1.02 (0.92–1.13)	0.82* (0.67–0.99)
Child’s size at birth			
Large	1	1	1
Average	0.81*** (0.76–0.86)	0.74*** (0.67–0.81)	0.68*** (0.57–0.82)
Small	1.15** (1.06–1.26)	1.03 (0.91–1.16)	0.87 (0.69–1.09)
Birth order			
1^st^ order	1	1	1
2^nd^ order	1.16*** (1.07–1.25)	1.02 (0.91–1.15)	1.07 (0.85–1.34)
3^rd^ order and above	1.24*** (1.11–1.39)	1.31** (1.12–1.55)	1.16 (0.86–1.58)
Place of delivery			
Outside health facility	1	1	1
Health facility	1.02 (0.94–1.10)	0.94 (0.84–1.05)	0.87 (0.70–1.08)
Source of water			
Not improved	1	1	1
Improved	0.93* (0.87–0.99)	1.00 (0.92–1.10)	1.07 (0.90–1.27)
Method of cooking			
Not improved	1	1	1
Improved	0.94 (0.73–1.21)	0.68 (0.44–1.06)	0.47 (0.11–1.94)

Level of significance at

*p<0.05,

**p<0.01,

***p<0.001

## Discussion

This study has revealed the magnitude of childhood morbidity in Nigeria and the factors that are associated with it. It shows that children in the country are not only exposed to the risk of single health condition, they are also exposed to the risk of comorbidity. Findings from the study show that children of young women (15–24 years) are more likely to suffer from morbidity than children of women of middle age (25–34 years) and aged 35 years and above. This could be linked to the fact that older women have accumulated experience over the years on issues relating to child health, particularly those that constitute preventive measures. Young women are considered to be relatively new in child care practices and as a result lack such experience. Contrary to expectation, education does not really show an inverse relationship with childhood morbidity as children of women with no education are less likely to experience morbidity when compared to children of educated women [20, 21]. There may have been some interplay of other factors which give the uneducated women an advantage over their counterparts. For instance, the uneducated women may have been exposed to some health care awareness programmes which promote sound knowledge about hygiene and prevention of childhood diseases which consequently improved their knowledge about child health [22]. Poverty at household level contributes significantly to morbidity among children. Both single health condition and comorbidity are more prevalent among children from poor households compared to children from rich households [23–27]. Lack of money could expose children to the risk of diseases in different ways. Living in slums or dirty environment due to poverty may predispose children to diseases such as diarrhoea. Since children need nutritious food to build their immunity which helps in fighting diseases, lack of money for procurement of nutritious food may force households to feed children with whatever is available which may compromise their immunity. Lack of money particularly for transportation may also prevent mothers from accessing facility where information on effective child care practices could be easily obtained or where immunization aimed at preventing diseases is administered. Geographical location is another factor that influences morbidity among children [28, 29]. Northeast, Southeast and South-south are the three regions with high propensity for childhood morbidity in the country. This also lends credence to childhood mortality in the sense that Northeast and Southeast have relatively high under-five mortality. The age of a child plays an important role in childhood morbidity [30–33]. Children who are less than one year and those who are between 1 and 2 years of age experience morbidity more than children who are three years and above. This is in consonance with the fact that such children are at risk of diseases because of their low immunity. Our findings further show that the size of a child at birth is significantly associated with childhood morbidity. While children of average size have lower odds of experiencing morbidity, children of small size or large size are more likely to experience morbidity. In the case of small size children, it has been observed that the smaller a child becomes, the more the risk of experiencing morbidity. This problem may be traced to the dietary intake of the mothers while in pregnancy. As reported in previous studies [34], small children are considered too fragile and as such, mothers would not present them for immunization. Birth order exerts some influence on childhood morbidity [35]. Children of second and higher birth order are more likely to experience morbidity compared to children of first order. This may be attributed to the fact that children of first order enjoy adequate attention and care from their mothers. As more children are born, such attention reduces because mothers need to share their time to take care of two or more children. Source of drinking water is significantly related to childhood morbidity. Children whose mothers obtain water from improved source are less prone to morbidity compared to children whose mothers obtain water from non-improved source. Getting water from non-improved source increases the risk of contamination which eventually exposes children who consume such water to water-borne diseases [36–40].

### Policy implications

With 14% of children experiencing a single health condition and close to 10% experiencing comorbidity, the efforts at putting Nigeria on track of achieving sustainable development goal 3, which emphasises living healthy lives and promoting well-being, may be jeopardized. In order to ensure a reasonable reduction in under-five mortality, adequate attention should be given to childhood morbidity reduction. In this light, the current programmes being put in place to improve child health should be strengthened through a review of the disease prevention measures. For instance, efforts should be geared towards investigating the use of insecticide treated nets by those who have benefited from its distribution. Are the nets used for the purpose for which they have been distributed? Are mothers and caregivers really putting into practice what they have learned about hygiene? What mechanism should be adopted to ensure effective monitoring and evaluation of preventive intervention programmes? Since most young women lack experience in child care practices, efforts should be made by health workers to educate women during antenatal, delivery and postnatal care on childhood diseases prevention measures. Orientation programmes should also be organised at community level for those who do not visit health facility during pregnancy, delivery or postnatal period. At such gathering, emphasis should be placed on avoidance of therapies that may complicate the conditions of sick children. To make the programme all embracing, men should be actively involved. As a result of the impact of poverty on childhood morbidity, governments at federal and state levels should embark on poverty alleviation programmes to improve the financial status of household members. Such programmes should include provision of employment opportunities and empowerment of those who wish to engage in small and medium scale enterprises. In order to ensure that children consume nutritious food that would build their immunity, state governments should earmark funds for under-five feeding programme. Such programme should ensure distribution of food stuff to different communities. To also overcome the challenge of inability of mothers to reach health facility for child immunization, mobile immunization programmes should be encouraged. State governments in the regions where childhood morbidity is most prevalent should endeavour to evaluate their policies on child health and under-study other regions where morbidity is relatively low. Budgetary allocations on child health promotion should be reviewed upwardly. Overcoming the challenge of children being small at birth requires the cooperation of health workers. Pregnant women who attend antenatal clinic should be reminded at every visit of the required dietary intake and implications of non-compliance on their babies. At the same time, efforts should be made, through awareness programmes, to increase the number of pregnant women that attend antenatal clinic. More so, the notion that children who are small at birth may not be strong enough to receive immunization should be discouraged. Mothers should be encouraged to pay adequate attention to their children irrespective of their birth order. Priority should also be given to provision of infrastructural facilities which would promote access to pure water and clean environment. This would go a long way in preventing children from diseases occasioned by poor water and dirty environment. There is a need for the government at all levels to develop a health care model which should be adequately implemented. The health care model should comprise, among other things, infrastructural development, regular feeding programme for children, community health awareness campaign and poverty alleviation initiatives

### Study strengths and weaknesses

It should be noted that findings from this study are based on analysis of data set from DHS. The survey obtained information about children’s characteristics from mothers and caregivers. The responses provided by the participants are subject to individual understanding and views about the phenomenon being investigated which may fall short of situation on the ground. Since questions about childhood morbidity related to past events, recall error might have occurred. Moreover, the hierarchical nature of the data lends credence to the application of multilevel modelling in order to measure the effects of contextual factors on childhood morbidity which this study has not applied. Lack of multilevel modelling hides structure in the data. In spite of these shortcomings, the survey is cross-sectional and provides adequate information that is nationally representative. The study has been able to apply generalized ordinal logistic regression model to capture the effects of independent variables on the ordered categories of the outcome variable. Results obtained from this study may be related to population with similar characteristics.

## Conclusions

The study has demonstrated that children in Nigeria are not only exposed to the risk of single health condition but they are also exposed to the risk of comorbidity with socioeconomic, demographic and environmental factors playing a significant role. Efforts should be made to design appropriate health care model that would facilitate a considerable reduction in childhood morbidity in the country

## List of abbreviations used

aOR: Adjusted Odds Ratio
CI: Confidence Interval
IHME: Institute of Health Metrics and Evaluation
OR: Odds Ratio
UNICEF: United Nations International Children’s Emergency Fund
WHO: World Health Organization
